# Radical Scavenging Activity of Puerarin: A Theoretical Study

**DOI:** 10.3390/antiox8120590

**Published:** 2019-11-26

**Authors:** Huakang Zhou, Xiangzhou Li, Yaxuan Shang, Kai Chen

**Affiliations:** 1 School of Materials Science and Engineering, Central South University of Forestry and Technology, Changsha 410004, China; zhouhuakang@126.com (H.Z.); 13808466928@163.com (X.L.); shang_yaxuan@163.com (Y.S.); 2 College of Chemistry and Chemical Engineering, Central South University, Changsha 410083, China; 3 State Key Laboratory of Chemical Oncogenomics, Peking University Shenzhen Graduate School, Shenzhen 518055, China

**Keywords:** puerarin, daidzein, antioxidant, DFT, radical scavenging activity

## Abstract

Puerarin is a C-glycoside of daidzein, one of the major bioactive ingredients isolated from the root of *Pueraria lobata*, which has a wide spectrum of pharmacological effects. Although puerarin is well-known for its effective antioxidant activity, there is seldom a systematic theoretical study on its radical scavenging activity. Herein, the free radical scavenging ability of puerarin was investigated systematically by density functional theory (DFT) calculations. The reaction activity was compared with daidzein as well. Three reaction pathways: hydrogen atom transfer (HAT), single electron transfer followed by proton transfer (SET-PT), and sequential proton loss electron transfer (SPLET) were discussed and compared by thermodynamic parameters such as bond dissociation enthalpy (BDE), ionization potential (IP), proton dissociation enthalpy (PDE), proton affinity (PA), and electron transfer enthalpy (ETE). The reaction kinetics of puerarin with special radicals •OH and •OOH were also studied. The results obtained may be of great significance for better understanding the relationship between the antioxidant properties and structural design of puerarin, as well as other antioxidants.

## 1. Introduction

Reactive oxygen species (ROS) are among the most harmful free radicals in the human body, such as hydroxyl radical (•OH), superoxide anion radical (•O^2−^), peroxyl radicals (ROO•), etc. [1,2]. If excessive ROS are produced in vivo, the antioxidant system will be out of balance, called oxidative stress, which could damage the structures and functions of the biological macromolecules such as lipids, proteins, RNA, and DNA [3,4]. Numerous human diseases are related to oxidative stress, like aging, atherosclerosis, diabetes, and Parkinson’s disease [5,6]. Synthetic antioxidants such as butylated hydroxyanisole (BHA), butylated hydroxytoluene (BHT), propyl gallate, and *tert*-butyl hydroquinone (TBHQ) are widely used as antioxidants or preservatives in foods, medicines, animal feeds, petroleum products, cosmetics, rubbers, etc. However, synthetic antioxidants often cause toxic and carcinogenic problems [7,8]. Hence, the development of more efficient, less toxic, and safer natural antioxidants has attracted broad interest.

**Figure 1:**
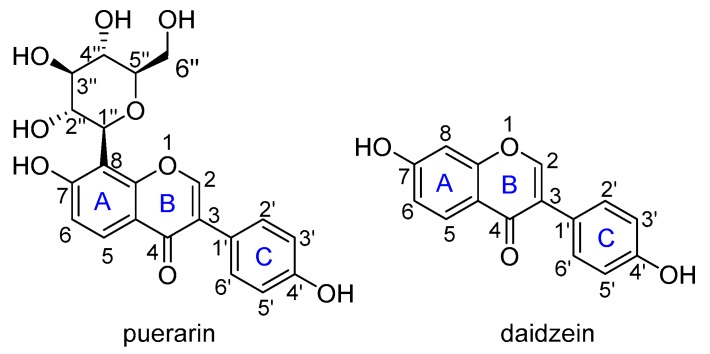
The molecular structure of puerarin and daidzein.

Flavonoids are reported to be important natural antioxidants from plants, which have plenty of pharmacological properties such as antioxidant, anticancer, anti-inflammation, vasodilation, alleviating pain, etc., [9,10]. Puerarin (4’,7-dihydroxy-8-β-d-glucosyliso-flavone) is a C-glycoside of daidzein, easily soluble in water with a solubility of 1.1 × 10^−2^ M (Figure 1) [11]. It is a major isoflavone extract from *P. lobata* roots, has potent antioxidant properties by scavenging free radicals and increasing the activity of superoxide dismutase as well [12]. Cheng et al. found that puerarin could significantly reverse H_2_O_2_-induced oxidative stress injury and decrease ROS production [13]. Bebrevska et al. evaluated the antioxidant activity of *P. lobata* root extract and found it was very efficient and safe in vivo [14]. Tian et al. examined the dynamics of excited states and radicals of puerarin by means of laser flash photolysis and spin-density analysis, which revealed the presence of long-lived puerarin radical surviving longer than milliseconds [15]. Yi and co-workers performed a complete NMR analysis of puerarin and daidzein, and explored the antioxidative activity by bond dissociation enthalpy (BDE) calculations [16]. However, to the best of our knowledge, there is still no systematic theoretical study on the antioxidant activity of puerarin. Herein, a systematic theoretical study on the antioxidant activities of puerarin was carried out to understand the radical scavenging mechanism of this natural product. Considering the relationship between puerarin and daidzein, a comparative study was carried out to help understand the activity.

## 2. Computational Methods

Geometry optimization and frequency calculations were performed by M06-2X [17] functional in conjunction with 6-31G(d) basis set in gas phase. Then single point calculations were conducted at M06-2X/6-311++G(d,p) theoretical level in different environments, considering the physiological medium is water while the possible action site could be the lipid membrane. Truhlar’s solvation model based on density (SMD) was chosen to account for the solvation effect [18]. Unrestricted calculations were used for open shell systems. Local minima were confirmed without imaginary frequencies, while transition states have only one imaginary frequency. Intrinsic reaction coordinate (IRC) calculations were performed to guarantee the connections between each transition state and the designated local minima. All of the calculations were completed utilizing Gaussian16 code (RevA.03, Gaussian Inc., Wallingford CT, USA) [19]. Optimized structure and frontier molecular orbital plots were produced by applying CYLview2.0 (University of Sherbrooke, Quebec, Canada) [20] or GaussView6.0 (Semichem Inc., Shawnee Mission, Kansas, USA) [21].

Phenolic antioxidants could scavenge free radicals through three possible action mechanisms [22,23,24]: hydrogen atom transfer (HAT), single electron transfer followed by proton transfer (SET-PT), and sequential proton loss electron transfer (SPLET). For HAT mechanism, phenolic antioxidant (ArOH) reacts with a free radical (R•) by transferring a hydrogen atom to the free radical through homolytic rupture of the O–H bond. Then, the antioxidant reactivity of ArOH could be evaluated by BDE(ArO–H), which could be calculated as follows:

BDE(ArO–H) = H(ArO•) + H(H•) − H(ArOH)(1)

For the SET-PT mechanism, it involves two steps: electron transfer from ArOH to give radical cation (ArOH•^+^) followed by proton transfer from ArOH•^+^. The antioxidant activity could be described by the ionization potential (IP) and proton dissociation enthalpy (PDE) values:

IP = H(ArOH•^+^) + H(e^−^) − H(ArOH) (2)

PDE = H(ArO•) + H(H^+^) − H(ArOH•^+^) (3)

For the SPLET mechanism, it is initiated by proton loss to form anion (ArO^−^) and then undergoes electron transfer to give ArO•, which could be revealed by proton affinity (PA) and electron transfer enthalpy (ETE) values:

PA = H(ArO^−^) + H(H^+^) − H(ArOH) (4)

ETE = H(ArO•) + H(e^−^) − H(ArO^−^) (5)

Here H(H•), H(ArOH), H(ArO•), H(ArO^−^), and H(ArOH•^+^) are the enthalpies of hydrogen atom, antioxidant, neutral radical, anion, and radical cation, respectively. The enthalpies of proton H(H^+^) and electron H(e^−^) were obtained from literatures [25,26].

## 3. Results and Discussion

### 3.1. Stable Conformation

**Figure 2:**
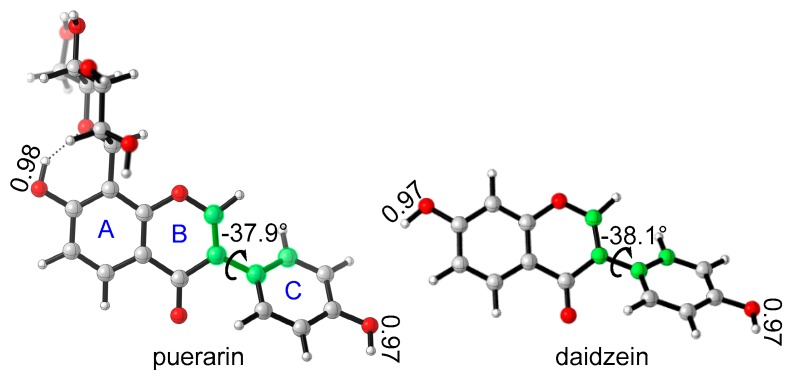
The stable conformations of puerarin and daidzein.

The most stable conformations of puerarin and daidzein were obtained after systematic conformational search. The isoflavone scaffold is close to a plane, and there is a small dihedral angle between chromenone and phenyl ring **C**, −37.9 °C for puerarin, or −38.1 °C for daidzein (Figure 2). In puerarin, the bond length of 7-O–H is a little longer than that of 4′-O–H, because of a weak intramolecular hydrogen bond between 7-OH and the glycosyl group. The glycosyl group is in chair conformation, where four hydroxyl groups form three intramolecular hydrogen bonds. Introduction of a glucose moiety makes puerarin strongly hydrophilic. For example, the solubility of puerarin in water, 1.1 × 10^−2^ M [11], is much better than that of daidzein, only 5.3 × 10^−6^ M [27], which makes puerarin’s oral bioavailability much better than daidzein [28].

### 3.2. Frontier Molecular Orbital Analysis

**Figure 3:**
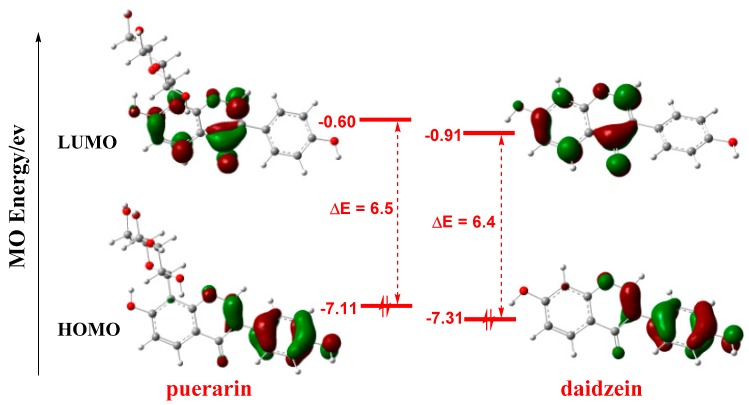
FMO of puerarin and daidzein. Isovalue for MO surface: 0.05, orbital energies in eV. FMO: frontier molecular orbital; MO: molecular orbital; HOMO: highest occupied molecular orbital; LUMO: lowest unoccupied molecular orbital.

Frontier molecular orbitals (FMO) play important roles in the reactivities of molecules [29,30,31]. As shown in Figure 3, FMO orbitals are not distributed on the glycosyl group of puerarin, and the orbital shapes of puerarin and daidzein are similar, which indicates the glycosyl group unlikely participated in the reaction. Thus, the glycosyl group is not considered in the following calculations. In both molecules, the highest occupied molecular orbital (HOMO) is mainly distributed on the phenyl ring **C**, while the lowest unoccupied molecular orbital (LUMO) largely lies on the chromenone moiety. As antioxidant mostly functioned as electron donor to provide electron to radical, the phenyl ring **C** should play a more important role in radical scavenging reactions. The HOMO-LUMO gaps were close, 6.5 eV and 6.4 eV for puerarin and daidzein, respectively, which were similar to that of another natural antioxidant, resveratrol, 6.3 eV [32].

### 3.3. HAT Mechanism

BDE(ArO–H) is one of the most important indicators to evaluate HAT mechanism (Table 1). BDE(4′-O–H) of puerarin and daidzein in water are 88.2 and 88.3 kcal/mol, respectively, a little lower than BDE(7-O–H), which indicates 4′-OH is the primary reaction site, similar to previous studies on other isoflavonoids [33,34]. BDE(O–H) of phenol has been determined by several experiments, and the recommended value is ~88.7 kcal/mol [35,36], very close to BDE(4′-O–H) here. A stronger bond of 7-OH than 4′-OH could be explained by the electron-withdrawing effect of pyrone moiety, which leads to puerarin-7-O• radical less stable than puerarin-4′-O• radical. BDE in polar environment is a little larger than that in non-polar solvent. However, the difference of BDE values among different solvents is still within 3.0 kcal/mol, similar to previous studies on other similar polyphenols [33,37,38]. As the glucose moiety sits far away from the reaction site, the antioxidant activities of puerarin and daidzein should be similar, which was also supported by the values of BDE(4′-O–H).

### 3.4. SET-PT Mechanism

In SET-PT mechanism, an electron is first transferred from natural antioxidant (ArOH) to a free radical leading to the formation of cation radical (ArOH•^+^), then a proton is transferred from ArOH•^+^ to give ArO•, described by IP and PDE, respectively. Different from BDE values, the solvent polarity has significant effects on the IP and PDE values (Table 2), which could be attributed to the high solvation enthalpies of proton and electron [39,40]. The IP values of puerarin in gas phase, benzene, and water are 179.9, 154.7, and 113.3 kcal/mol, respectively. The sequence of PDEs in different solvents are analogous with those of IPs and the lowest PDE is in water. The IP values of daidzein are close to those of puerarin. As IP values are larger than PDE values in solvent, the first step is thermodynamically significant for SET-PT mechanism. For both molecules, IPs in solvent are at least 25 kcal/mol higher than the lowest BDE, which indicates the SET-PT pathway is not as favorable as the HAT pathway. This conclusion is similar to the references, which confirms the accuracy of this work [33,37,38].

### 3.5. SPLET Mechanism

SPLET mechanism also plays an important role in free radical scavenging reactions (Table 3). The calculated PAs and ETEs are listed in Table 3. Similar to PDEs, PA values show a significant decrease from gas phase to solvent, which could be explained by the large solvation enthalpies of proton and anion. For both molecules, in gas phase and non-polar solvent, PA(7-OH) is larger than ETE(4′-OH), while in polar solvents the former is lower than the latter. It means that in water, the determining step in SPLET is the second step thermodynamically. As ETE(4′-OH) is even lower than BDE(4′-OH), SPLET pathway is more favorable than HAT pathway in water. However, in non-polar solvent, the smaller PA values, PA(7-OH) are larger than BDE(4′-OH), thus HAT should be the dominant pathway. These results agree well with the previous studies of flavonoids and isoflavonoids [33,37,38].

The above analysis suggested that in water SPLET pathway is dominant, while in non-polar solvent, HAT pathway is preferred. As in water, the determining step of SPLET pathway is the second step thermodynamically, ETE values of the reaction site, 4′-OH, are close, 82.1 and 83.4 kcal/mol for puerarin and daidzein, respectively, while in non-polar solvent, BDE values of the reaction site, 4′-OH, are 86.6 and 86.4 kcal/mol for puerarin and daidzein, respectively. Thus, puerarin and daidzein have very similar antioxidant activity, which was also supported by experimental results [27,41]. Considering the solubility and bioavailability of puerarin is much better than daidzein, puerarin should have a better antioxidant.

### 3.6. Kinetics of Free Radical Scavenging by Puerarin

To have a better understanding of the radical scavenging properties of puerarin, the kinetics of puerarin with representative free radicals was studied. Hydroxyl radical (•OH) and hydroperoxyl radical (•OOH) were chosen as the two representative ROS, which reacted with puerarin as following:

Puerarin-OH + •OH → Puerarin -O• + H_2_O (6)

Puerarin-OH + •OOH → Puerarin -O• + H_2_O_2_ (7)

**Figure 4:**
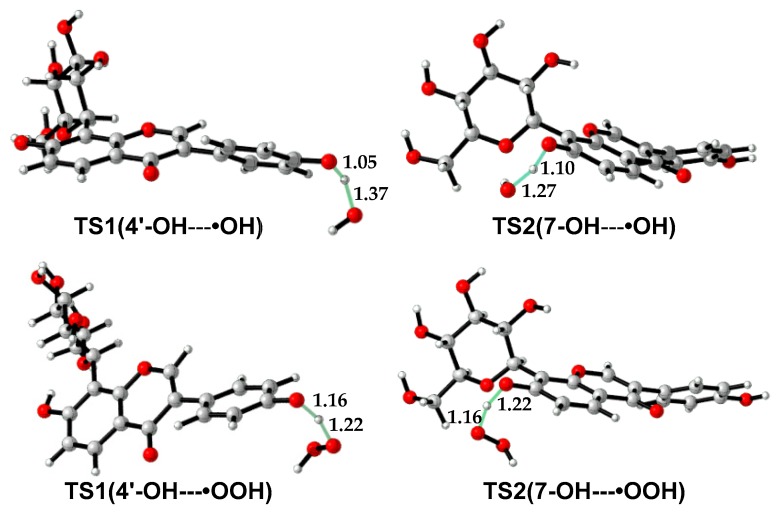
Transition state structures in the reactions of puerarin with radical •OH or •OOH.

As •OH is much more reactive than •OOH, the reaction barrier of puerarin with •OH is much lower than that with •OOH (Table 4, Table S1). In the molecule, 4′-OH is more reactive than 7-OH. For example, in water, the free energy barrier of 4′-OH with •OH is 12.2 kcal/mol lower than that of 7-OH with •OH. The former is as low as 3.0 kcal/mol, which suggests a diffusion-controlled process, according to the transition state theory [42,43]. Although the reaction barriers are less than 20 kcal/mol in water, the reactions with •OOH are endergonic except 4′-OH with •OOH, which suggests puerarin is not an efficient scavenger of •OOH. Transition state structures are shown in Figure 4. In TS(4′-OH---•OH), the forming and breaking O—H bonds are 1.37 and 1.05 Å in length, while in TS(4′-OH---•OOH), the forming and breaking O—H bonds are 1.22 and 1.16 Å in length. The forming O-H bond in TS(4′-OH---•OH) or TS(7-OH---•OH) is longer than that in the corresponding transition structure with •OOH, indicating the former is an earlier transition state than the latter.

## 4. Conclusions

The radical scavenging activity of puerarin was investigated under the theoretical level of M062X/6-311++G(d,p)//M062X/6-31G(d). Three reaction mechanisms were considered: HAT, SET-PT, and SPLET. It reveals that HAT should be the preferred mechanism in non-polar solvents, while SPLET would be more favorable in polar media, thermodynamically. The reaction activity was compared with daidzein, which shows puerarin and daidzein have very similar antioxidant activity. However, as puerarin has better solubility and bioavailability, puerarin should be a better antioxidant than daidzein. The reaction kinetics of puerarin with •OH or •OOH radicals were also investigated. The reactions with •OH have much lower energy barrier than those with •OOH. All these results suggests 4′-OH is perhaps the most reactive site to scavenge radicals, which agrees well with previous studies. These results might be helpful for interpreting puerarin’s antioxidant activity and for further designing new potential derivatives.

## Figures and Tables

**Table 1:** The O–H BDEs of puerarin and daidzein in gas phase and different solvents (Units: kcal/mol).

Sites	Gas	Benzene	Water
puerarin			
4′-OH	87.3	86.6	88.2
7-OH	97.0	96.5	96.8
daidzein			
4′-OH	86.9	86.4	88.3
7-OH	91.6	91.7	95.3

**Table 2:** Ionization potentials (IPs) and proton dissociation enthalpies (PDEs) of puerarin and daidzein in gas phase and different solvents.

Sites	IP (kcal/mol)			PDE (kcal/mol)		
	Gas	Benzene	Water	Gas	Benzene	Water
puerarin	179.9	154.7	113.3			
4′-OH				220.8	29.8	17.6
7-OH				230.5	39.7	26.1
daidzein	178.9	153.9	113.4			
4′-OH				213.0	21.9	9.1
7-OH				217.4	27.0	15.8

**Table 3:** Proton affinities (PAs) and electron transfer enthalpies (ETEs) of puerarin and daidzein in gas phase and different solvents.

Sites	PA (kcal/mol)			ETE (kcal/mol)		
	Gas	Benzene	Water	Gas	Benzene	Water
puerarin						
4′-OH	338.0	103.0	48.8	62.7	81.4	82.1
7-OH	317.0	87.3	40.8	93.4	107.1	98.7
daidzein						
4′-OH	332.2	95.0	39.1	59.7	80.8	83.4
7-OH	319.9	85.1	32.9	76.4	95.7	96.3

**Table 4:** Free energy barriers for the reaction of puerarin with •OH or •OOH in gas phase and solution.

Sites	ΔG^≠^(kcal/mol)			ΔG (kcal/mol)		
	Gas	Benzene	Water	Gas	Benzene	Water
Reaction with •OH						
4′-OH	6.8	8.0	3.0	−29.7	−31.2	−33.0
7-OH	10.1	11.8	15.2	−20.9	−22.2	−25.3
Reaction with •OOH						
4′-OH	16.6	18.1	19.4	1.8	0.9	−0.5
7-OH	22.4	25.4	26.8	10.6	9.9	7.1

## Supplementary Materials

The following are available online at https://www.mdpi.com/2076-3921/8/12/590/s1, Table S1: Thermal energies of all stationary points and Cartesian coordinates for all stationary points.
