# Supplementary material for: Radical Scavenging Activity of Puerarin: A Theoretical Study
Source: Antioxidants (Basel). 2019 Nov 26;8(12):590. doi: 10.3390/antiox8120590 (PMC6943502; doi:10.3390/antiox8120590)
Supplement: Supplementary file 1 [file antioxidants-08-00590-s001.pdf]

Electronic Supplementary Information

Radical Scavenging Activity of Puerarin: A Theoretical Study

Huakang Zhou[1], Xiangzhou Li[1], Yaxuan Shang[1], and Kai Chen[2,3,*]

[1] School of Materials Science and Engineering, Central South University of Forestry and Technology, Changsha 410004, P. R. China.

[2] College of Chemistry and Chemical Engineering, Central South University, Changsha 410083, P. R. China

[3] State Key Laboratory of Chemical Oncogenomics, Peking University Shenzhen Graduate School, Shenzhen 518055, P. R. China.

Correspondence: cekchen@scut.edu.cn (K.C.)

Table S1. Thermal energies of all stationary points.

**Species**	E_{ele}	E_0	E	H	G	E_{ele}(gas)	E_{ele}(ben)	E_{ele}(wat)
Puerarin	-1488.67784	-1488.28077	-1488.25505	-1488.25410	-1488.33662	-1489.14536	-1489.17559	-1489.19382
Puerarin cation radical	-1488.40329	-1488.00609	-1487.98019	-1487.97925	-1488.06302	-1488.86016	-1488.92797	-1488.97474
Puerarin-4'-O^- anion	-1488.11763	-1487.73444	-1487.70893	-1487.70798	-1487.79036	-1488.59503	-1488.65915	-1488.71499
Puerarin-4'-O• radical	-1488.03615	-1487.65228	-1487.62681	-1487.62587	-1487.70875	-1488.49697	-1488.52854	-1488.54605
Puerarin-7-O^- anion	-1488.15499	-1487.77111	-1487.74611	-1487.74517	-1487.82598	-1488.62862	-1488.68443	-1488.72806
Puerarin-7-O• radical	-1488.01879	-1487.63615	-1487.61017	-1487.60923	-1487.69359	-1488.48076	-1488.51205	-1488.53170
daidzein	-878.156087	-877.936105	-877.921473	-877.920529	-877.978106	-878.408222	-878.430274	-878.435672
Daidzein cation radical	-877.884115	-877.663587	-877.649015	-877.648071	-877.706368	-878.125341	-878.184521	-878.217057
Daidzein -4'-O^- anion	-877.590604	-877.384515	-877.370087	-877.369143	-877.426455	-877.853076	-877.912479	-877.958192
Daidzein -4'-O• radical	-877.51507	-877.308221	-877.293841	-877.292897	-877.350815	-877.760479	-877.783739	-877.787908
Daidzein -7-O^- anion	-877.611441	-877.404926	-877.390532	-877.389588	-877.44678	-877.873457	-877.929082	-877.968893
Daidzein -7-O• radical	-877.507335	-877.300982	-877.286555	-877.285611	-877.343652	-877.752633	-877.774778	-877.776328
H_2O	-76.37340	-76.35187	-76.34903	-76.34809	-76.36952	-76.42082	-76.42535	-76.43476
H_2O_2	-151.46819	-151.44112	-151.43791	-151.43696	-151.46274	-151.53519	-151.54121	-151.55100
OH• radical	-75.68866	-75.68017	-75.67781	-75.67687	-75.69711	-75.72658	-75.73015	-75.73596
OOH• radical	-150.83272	-150.81815	-150.81530	-150.81435	-150.84030	-150.89052	-150.89637	-150.90318
TS(puerarin_4'-OH---•OH)	-1564.36826	-1563.96451	-1563.93656	-1563.93562	-1564.02450	-1564.87203	-1564.90392	-1564.93598
TS(puerarin_7-OH---•OH)	-1564.36213	-1563.95962	-1563.93177	-1563.93083	-1564.01922	-1564.86590	-1564.89699	-1564.91569
TS(puerarin_4'-OH---•OOH)	-1639.49690	-1639.08866	-1639.05987	-1639.05892	-1639.15080	-1640.02182	-1640.05558	-1640.07849
TS(puerarin_7-OH----•OOH)	-1639.48532	-1639.07777	-1639.04877	-1639.04783	-1639.13861	-1640.01027	-1640.04318	-1640.06827

Notes: E_{ele}: electronic energy, E_0 = E_{ele} + Zero-point correction, E = E_{ele} + thermal correction to Energy, H = E_{ele} + thermal correction to Enthalpy, and G = E_{ele} + thermal correction to Gibbs free energy are calculated at M06-2X/6-31G(d) theoretical level; E_{ele}(gas), E_{ele}(ben), and E_{ele}(wat) are electronic energies in gas phase, benzene, and water, respectively, at M06-2X/6-311++G(d,p) theoretical level.

Cartesian coordinates for all stationary points.

H_2O

O	0.000000	0.000000	0.118474
H	0.000000	0.762375	-0.473898
H	-0.000000	-0.762375	-0.473898

H_2O_2

O	-0.000000	-0.713662	-0.055435
H	-0.813023	-0.891365	0.443482
O	0.000000	0.713662	-0.055435
H	0.813023	0.891365	0.443482

HO•

O	-0.000000	-0.000000	0.108762
H	0.000000	0.000000	-0.870096

HOO•

O	0.055510	-0.601672	0.000000
H	-0.888157	-0.863496	0.000000
O	0.055510	0.709609	0.000000

TS(puerarin_4'-OH---•OH)

C	1.524676	1.084070	-0.230522
C	0.139893	0.894777	-0.123971
C	1.987401	2.400098	-0.360076
C	-0.765888	1.954775	-0.134739
C	1.090805	3.484678	-0.371176
C	-1.613346	-0.637920	0.097725
C	-2.216291	1.717359	-0.019530
C	-0.261574	3.259039	-0.256622
H	1.502007	4.482072	-0.477365
C	-2.594108	0.289117	0.070626
H	-1.797619	-1.700374	0.211850
H	-0.977685	4.074352	-0.263364
O	3.299580	2.689221	-0.458968
H	3.777976	1.874272	-0.709734
O	-0.290189	-0.393627	0.009206
C	-4.008931	-0.129074	0.133928
C	-4.435367	-1.289317	-0.529360
C	-4.952006	0.611445	0.866961
C	-5.745509	-1.730412	-0.438703
H	-3.730363	-1.844462	-1.141959
C	-6.269643	0.196749	0.943201
H	-4.642383	1.524093	1.360777
C	-6.675250	-0.987686	0.308185
H	-6.072450	-2.627688	-0.954667
H	-7.009768	0.761688	1.499574
O	-7.946030	-1.383633	0.452122
H	-8.227045	-1.964270	-0.371646
O	-3.024540	2.632037	-0.002109
C	2.478701	-0.075109	-0.096424
C	3.045905	-0.152573	1.332955
C	4.050370	-1.286243	1.414012
C	5.121730	-1.091875	0.358998
C	4.480238	-0.991696	-1.023780
H	1.958004	-1.016650	-0.313742
O	3.554378	0.093455	-1.023206
C	5.502324	-0.734876	-2.127281
H	4.966373	-0.477182	-3.044726
H	6.122720	0.131900	-1.844713
O	6.270776	-1.878002	-2.394851
H	6.652962	-2.174296	-1.552800
H	3.949300	-1.928607	-1.249883
O	6.034260	-2.169442	0.362718
H	6.301411	-2.305257	1.286434
H	5.643674	-0.142221	0.566828
O	4.700479	-1.333853	2.663823
H	4.007884	-1.384210	3.341028
H	3.523201	-2.233925	1.212358
H	3.559366	0.794869	1.561144
O	2.042444	-0.433654	2.282982
H	1.478954	0.349046	2.375953
O	-8.642491	-2.031399	-1.675748
H	-8.280621	-1.233915	-2.105553

TS(puerarin_4'-OH---•OOH)

C	1.773235	1.085993	-0.185013
C	0.393646	0.881400	-0.049021
C	2.220303	2.409485	-0.295428
C	-0.522369	1.932027	-0.013371
C	1.313329	3.485031	-0.260021
C	-1.337172	-0.675030	0.182962
C	-1.966646	1.677463	0.132034
C	-0.033628	3.243742	-0.118061
H	1.712523	4.488568	-0.353299

C	-2.327129	0.243604	0.207244
H	-1.508514	-1.741517	0.278291
H	-0.757098	4.052007	-0.089140
O	3.526213	2.714203	-0.419295
H	4.007098	1.909718	-0.698062
O	-0.021318	-0.414861	0.064192
C	-3.732807	-0.190973	0.311231
C	-4.167726	-1.348590	-0.354154
C	-4.655737	0.534244	1.094418
C	-5.469662	-1.797073	-0.226544
H	-3.479073	-1.883206	-1.002217
C	-5.959042	0.101723	1.220638
H	-4.330546	1.441098	1.588942
C	-6.393420	-1.083282	0.577200
H	-5.816330	-2.686657	-0.741286
H	-6.680109	0.642300	1.824880
O	-7.627019	-1.503524	0.734537
H	-8.196721	-1.441294	-0.269113
O	-2.784786	2.582212	0.180959
C	2.741213	-0.066639	-0.102188
C	3.341889	-0.174824	1.311448
C	4.359128	-1.299879	1.340074
C	5.403489	-1.067434	0.265770
C	4.729052	-0.938621	-1.098807
H	2.224890	-1.007738	-0.331590
O	3.792868	0.136880	-1.048695
C	5.722644	-0.643576	-2.218675
H	5.163066	-0.366828	-3.116239
H	6.342286	0.220998	-1.927849
O	6.493863	-1.772369	-2.534189
H	6.899811	-2.086277	-1.709835
H	4.201674	-1.874559	-1.336758
O	6.326224	-2.135123	0.220266
H	6.617302	-2.291411	1.133467
H	5.920824	-0.118037	0.485965
O	5.038806	-1.372108	2.572694
H	4.363286	-1.449024	3.264479
H	3.836830	-2.247603	1.126384
H	3.851418	0.771570	1.552357
O	2.362852	-0.490133	2.276147
H	1.797462	0.285893	2.405879
O	-8.601412	-1.029957	-1.341065
O	-7.435363	-0.795753	-2.011721
H	-7.333055	0.173528	-1.966315

TS(puerarin_7-OH---•OH)

C	0.991776	0.947899	0.133172
C	-0.389504	0.711170	0.102212
C	1.418152	2.283281	0.037943
C	-1.319601	1.744961	-0.030921
C	0.492090	3.334532	-0.121086
C	-2.117853	-0.864465	0.165060
C	-2.773094	1.462547	-0.058787
C	-0.857727	3.063644	-0.148973
H	0.877102	4.345345	-0.199979
C	-3.117999	0.029358	0.015671
H	-2.282844	-1.930253	0.275996
H	-1.600521	3.847236	-0.256643
O	2.727484	2.574781	0.158051
H	3.155402	2.758347	-0.834824
O	-0.797980	-0.583060	0.209682
C	-4.520299	-0.429771	-0.073001
C	-4.842625	-1.604430	-0.766084
C	-5.556138	0.281151	0.543170
C	-6.145152	-2.075633	-0.823992
H	-4.061557	-2.146739	-1.292415
C	-6.864854	-0.180400	0.486861
H	-5.335494	1.205095	1.063562
C	-7.163662	-1.361555	-0.192498
H	-6.397750	-2.980145	-1.366352
H	-7.659453	0.381431	0.972569
O	-3.595806	2.360007	-0.137044
C	1.981569	-0.184319	0.244108
C	2.792829	-0.121890	1.550968
C	3.825924	-1.230863	1.546580
C	4.682006	-1.112896	0.301534
C	3.796106	-1.197351	-0.939435
H	1.439878	-1.139520	0.241904
O	2.841432	-0.141012	-0.894025
C	4.590737	-1.040461	-2.233715
H	3.884596	-0.921283	-3.061865
H	5.194130	-0.119969	-2.169015
O	5.366803	-2.176388	-2.509078
H	5.909858	-2.350644	-1.723051
H	3.281637	-2.170887	-0.956421
O	5.640857	-2.149853	0.251874
H	6.066559	-2.175188	1.124238
H	5.172208	-0.125283	0.304719

O	4.688314	-1.154514	2.660020
H	4.125340	-1.145250	3.449703
H	3.303787	-2.202605	1.527305
H	3.308775	0.842571	1.618820
O	1.961955	-0.348203	2.674108
H	1.458405	0.459740	2.849218
O	3.651114	2.316028	-1.912863
H	3.308473	1.395365	-1.923155
O	-8.425167	-1.859259	-0.281899
H	-9.036666	-1.262629	0.172353

TS(puerarin_7-OH----•OOH)

C	-0.939699	0.748143	-0.393571
C	0.440429	0.552874	-0.311113
C	-1.403960	2.057808	-0.697478
C	1.355684	1.598254	-0.481145
C	-0.472345	3.125779	-0.852400
C	2.195750	-0.946806	0.067369
C	2.812652	1.359055	-0.373829
C	0.876284	2.893155	-0.750445
H	-0.876963	4.107553	-1.074762
C	3.181201	-0.029946	-0.042252
H	2.380213	-1.997754	0.259918
H	1.611926	3.681126	-0.874404
O	-2.678698	2.295227	-0.864915
H	-3.211445	2.347494	0.232558
O	0.873089	-0.713023	-0.051511
C	4.590386	-0.422009	0.170306
C	4.928994	-1.351123	1.163491
C	5.617221	0.106091	-0.620324
C	6.238712	-1.765548	1.349818
H	4.154644	-1.738489	1.820365
C	6.932982	-0.299592	-0.438347
H	5.383757	0.844309	-1.377816
C	7.248113	-1.239232	0.543356
H	6.503607	-2.478854	2.122555
H	7.720440	0.117426	-1.062027
O	8.516965	-1.671445	0.767069
H	9.120243	-1.218359	0.161252
O	3.619171	2.258274	-0.550442
C	-1.917426	-0.382133	-0.208233
C	-2.595658	-0.766228	-1.537572
C	-3.631088	-1.839373	-1.261174
C	-4.603474	-1.347883	-0.207045
C	-3.839917	-0.964500	1.059868
H	-1.384818	-1.270471	0.161212
O	-2.885114	0.031792	0.736606
C	-4.750294	-0.382668	2.137200
H	-4.123613	0.047821	2.922926
H	-5.339788	0.437606	1.697976
O	-5.557197	-1.372620	2.725423
H	-6.010555	-1.838414	2.004356
H	-3.341862	-1.861016	1.464755
O	-5.549724	-2.350600	0.107549
H	-5.896441	-2.678760	-0.737743
H	-5.105924	-0.444970	-0.591296
O	-4.380318	-2.159904	-2.413351
H	-3.741108	-2.392492	-3.104634
H	-3.114458	-2.734514	-0.874871
H	-3.098070	0.117772	-1.951148
O	-1.675210	-1.321488	-2.458297
H	-1.149375	-0.602655	-2.836998
O	-2.185166	2.488725	1.904162
H	-1.821322	3.377186	2.079785
O	-3.378593	2.759280	1.305103

Puerarin-4'-O^- anion

C	1.102334	1.096271	-0.118999
C	-0.280390	0.822855	-0.094685
C	1.505551	2.430993	-0.032701
C	-1.233925	1.835789	0.010119
C	0.559035	3.465522	0.065599
C	-1.986855	-0.783173	-0.103078
C	-2.685972	1.532256	0.020611
C	-0.784801	3.162028	0.086418
H	0.922921	4.485521	0.120276
C	-3.027491	0.093145	-0.090434
H	-2.115036	-1.857076	-0.103105
H	-1.543319	3.935033	0.160827
O	2.815443	2.787712	-0.021712
H	3.332450	2.038155	-0.371307
O	-0.647490	-0.471683	-0.142949
C	-4.401085	-0.380412	-0.189445
C	-4.701990	-1.569815	-0.897032
C	-5.484054	0.253495	0.468881
C	-5.964553	-2.107428	-0.934789
H	-3.905896	-2.052510	-1.466593
C	-6.752272	-0.273906	0.448745

H	-5.296199	1.174371	1.012301
C	-7.090570	-1.502965	-0.245033
H	-6.169840	-3.004833	-1.513943
H	-7.565000	0.228349	0.968551
O	-8.242687	-1.988350	-0.263545
O	-3.500041	2.438679	0.112321
C	2.094813	-0.036377	-0.112548
C	2.596428	-0.309375	1.316543
C	3.648295	-1.399653	1.285459
C	4.764587	-1.002989	0.343328
C	4.188803	-0.729107	-1.045704
H	1.620799	-0.948057	-0.498668
O	3.214660	0.301563	-0.944411
C	5.251034	-0.263785	-2.036789
H	4.751834	0.076330	-2.948067
H	5.784227	0.599188	-1.603513
O	6.126066	-1.303709	-2.395292
H	6.441024	-1.710061	-1.571384
H	3.722249	-1.647925	-1.432665
O	5.735412	-2.029344	0.255196
H	5.914415	-2.312733	1.166580
H	5.223590	-0.071884	0.716541
O	4.226370	-1.623905	2.555057
H	3.488263	-1.755876	3.171138
H	3.178005	-2.323040	0.908303
H	3.049071	0.616879	1.707680
O	1.574455	-0.777854	2.164190
H	0.847406	-0.134914	2.159387

Puerarin-4'-O• radical

C	1.085754	1.088682	-0.120025
C	-0.289971	0.825668	-0.110881
C	1.484546	2.430071	-0.042580
C	-1.249168	1.832806	-0.021491
C	0.533456	3.463209	0.050496
C	-1.965791	-0.804553	-0.192673
C	-2.688647	1.516841	-0.012728
C	-0.809170	3.163017	0.063529
H	0.896314	4.483283	0.104277
C	-2.997222	0.071263	-0.135088
H	-2.095779	-1.880128	-0.232594
H	-1.565514	3.938034	0.132554
O	2.781948	2.789471	-0.033046
H	3.314745	2.039707	-0.365101
O	-0.659717	-0.488610	-0.179487
C	-4.378536	-0.424773	-0.184423
C	-4.691311	-1.577686	-0.952652
C	-5.411525	0.216515	0.548501
C	-5.957453	-2.094036	-0.973939
H	-3.914129	-2.032680	-1.560582
C	-6.684500	-0.287067	0.544287
H	-5.174660	1.109747	1.112065
C	-7.030142	-1.476413	-0.213223
H	-6.211999	-2.966321	-1.566936
H	-7.483122	0.181807	1.109809
O	-8.185132	-1.941613	-0.218513
O	-3.540741	2.384836	0.080673
C	2.090914	-0.034317	-0.097308
C	2.589949	-0.290695	1.336551
C	3.643938	-1.381440	1.313417
C	4.754586	-0.993645	0.356648
C	4.177579	-0.722274	-1.031587
H	1.630641	-0.956980	-0.473980
O	3.200230	0.312390	-0.928974
C	5.239159	-0.268444	-2.029366
H	4.737325	0.097897	-2.928944
H	5.804172	0.571789	-1.592663
O	6.071584	-1.330291	-2.413982
H	6.429317	-1.727018	-1.603143
H	3.704362	-1.637811	-1.417000
O	5.716826	-2.022001	0.257888
H	5.947668	-2.276500	1.166175
H	5.218955	-0.063039	0.725166
O	4.234037	-1.578890	2.577909
H	3.513652	-1.758886	3.201915
H	3.174660	-2.311962	0.952060
H	3.044428	0.635097	1.723246
O	1.554875	-0.745147	2.179772
H	0.959023	-0.003555	2.362956

Puerarin-7-O$^-$ anion

C	1.353537	1.487814	-0.018344
C	-0.007043	1.186339	0.001488
C	1.755404	2.823799	-0.377904
C	-1.026457	2.077391	-0.364970
C	0.695035	3.723484	-0.782920
C	-1.622224	-0.450777	0.465204
C	-2.428043	1.704917	-0.330771

C	-0.621433	3.369069	-0.775253
H	1.014908	4.717237	-1.081796
C	-2.677614	0.305830	0.096838
H	-1.720111	-1.464378	0.842514
H	-1.403376	4.062138	-1.072456
O	2.952228	3.250987	-0.351849
O	-0.337604	-0.077485	0.438205
C	-4.042094	-0.261362	0.139625
C	-4.275398	-1.605663	-0.182387
C	-5.142778	0.518380	0.518139
C	-5.542240	-2.169117	-0.098735
H	-3.447829	-2.218948	-0.528617
C	-6.415791	-0.034227	0.596457
H	-4.990492	1.568201	0.734339
C	-6.619205	-1.379327	0.294581
H	-5.717546	-3.209327	-0.352885
H	-7.259622	0.586384	0.893842
O	-7.854207	-1.967189	0.357302
H	-8.496759	-1.292552	0.616455
O	-3.345231	2.474397	-0.627140
C	2.299698	0.368418	0.340915
C	3.795023	0.707127	0.381095
C	4.549537	-0.542758	0.795925
C	4.264926	-1.672562	-0.165481
C	2.752945	-1.895745	-0.239586
H	2.017844	-0.037948	1.328607
O	2.134621	-0.689869	-0.624705
C	2.373260	-2.954615	-1.268150
H	1.283795	-2.970400	-1.364241
H	2.790094	-2.659473	-2.244798
O	2.788975	-4.247988	-0.885304
H	3.708214	-4.166738	-0.581774
H	2.395428	-2.225157	0.751938
O	4.916738	-2.867732	0.251749
H	5.822633	-2.605410	0.480543
H	4.618332	-1.378871	-1.166869
O	5.945622	-0.309115	0.821973
H	6.020361	0.593650	1.179109
H	4.201192	-0.843057	1.799883
H	4.099444	0.986293	-0.638630
O	4.142666	1.708502	1.297893
H	3.798757	2.523807	0.825231

Puerarin-7-O• radical

C	1.133831	1.068328	-0.140483
C	-0.227372	0.800930	-0.092562
C	1.556783	2.429171	-0.455741
C	-1.194282	1.783914	-0.365001
C	0.533188	3.419163	-0.762451
C	-1.917946	-0.782003	0.256043
C	-2.639472	1.462422	-0.311115
C	-0.788308	3.098760	-0.707695
H	0.884844	4.413978	-1.013181
C	-2.945517	0.053338	-0.010455
H	-2.054526	-1.820540	0.535955
H	-1.573688	3.817635	-0.915726
O	2.760532	2.750156	-0.432824
O	-0.607304	-0.468835	0.228394
C	-4.335787	-0.447509	0.000271
C	-4.634212	-1.725445	-0.492367
C	-5.382653	0.325803	0.514514
C	-5.923415	-2.232741	-0.451657
H	-3.845743	-2.324554	-0.940121
C	-6.678492	-0.172142	0.555287
H	-5.181783	1.325671	0.879407
C	-6.953121	-1.453561	0.077001
H	-6.157713	-3.218114	-0.839169
H	-7.481891	0.439245	0.959750
O	-8.200464	-1.991291	0.089520
H	-8.822100	-1.349589	0.461106
O	-3.481905	2.324370	-0.505833
C	2.166587	0.005711	0.119905
C	3.001631	0.299145	1.382023
C	4.040689	-0.795782	1.528535
C	4.864473	-0.888167	0.259401
C	3.944851	-1.138391	-0.935726
H	1.657288	-0.955314	0.284020
O	3.002420	-0.080945	-1.019953
C	4.707933	-1.182155	-2.256082
H	3.984071	-1.168678	-3.075286
H	5.325559	-0.274031	-2.339178
O	5.464907	-2.360158	-2.380746
H	6.013787	-2.433166	-1.583244
H	3.431007	-2.104379	-0.797067
O	5.803340	-1.942492	0.350368
H	6.262321	-1.831773	1.198525
H	5.374486	0.076109	0.103609
O	4.929321	-0.541398	2.594613

H	4.383332	-0.407001	3.384733
H	3.519684	-1.756405	1.681505
H	3.509337	1.262805	1.262542
O	2.209679	0.263654	2.556229
H	1.751188	1.110879	2.643817

Puerarin

C	1.133064	1.082216	-0.130203
C	-0.244113	0.817938	-0.101032
C	1.534161	2.423506	-0.093863
C	-1.199976	1.830588	-0.030321
C	0.585896	3.460544	-0.020440
C	-1.928723	-0.805107	-0.122080
C	-2.640654	1.516488	-0.000952
C	-0.756749	3.161584	0.013874
H	0.950534	4.481225	0.001350
C	-2.951968	0.073100	-0.091809
H	-2.064423	-1.880678	-0.135953
H	-1.512280	3.938621	0.069192
O	2.834018	2.781127	-0.107604
H	3.359547	2.022587	-0.429822
O	-0.613292	-0.493751	-0.127434
C	-4.347898	-0.410631	-0.150142
C	-4.691185	-1.497602	-0.965424
C	-5.356570	0.188017	0.613029
C	-5.985067	-1.994742	-1.002792
H	-3.934758	-1.947059	-1.603342
C	-6.656836	-0.299409	0.578424
H	-5.121324	1.046370	1.230222
C	-6.975006	-1.394173	-0.225229
H	-6.253379	-2.831064	-1.639013
H	-7.430064	0.175274	1.178382
O	-8.230432	-1.911553	-0.299384
H	-8.821845	-1.392311	0.263444
O	-3.490788	2.387938	0.093944
C	2.136701	-0.041296	-0.089104
C	2.659140	-0.254679	1.343311
C	3.711256	-1.347050	1.337068
C	4.807625	-0.990013	0.352888
C	4.208623	-0.761300	-1.033829
H	1.668459	-0.973627	-0.430361
O	3.234736	0.277034	-0.948392
C	5.254413	-0.339688	-2.061760
H	4.738514	-0.004396	-2.965509
H	5.823727	0.515774	-1.661647
O	6.084570	-1.411927	-2.423212
H	6.449098	-1.784891	-1.604105
H	3.729134	-1.688395	-1.382400
O	5.768392	-2.022085	0.272522
H	6.006278	-2.252461	1.185425
H	5.278089	-0.048911	0.685068
O	4.319391	-1.509129	2.598680
H	3.605413	-1.655462	3.238803
H	3.235219	-2.286963	1.010575
H	3.120453	0.682451	1.693447
O	1.639951	-0.682533	2.218650
H	1.024458	0.053200	2.356407

Puerarin cation radical

C	1.134803	1.114116	-0.096325
C	-0.235387	0.874346	-0.084953
C	1.539240	2.459404	0.022809
C	-1.201090	1.871203	0.041554
C	0.587278	3.488755	0.157647
C	-1.886038	-0.782954	-0.232121
C	-2.631026	1.554393	0.041213
C	-0.757992	3.197523	0.169077
H	0.952976	4.505576	0.244232
C	-2.952049	0.097611	-0.131267
H	-1.999179	-1.859150	-0.302558
H	-1.505008	3.978141	0.267021
O	2.824404	2.815545	0.037107
H	3.371225	2.068721	-0.287577
O	-0.623781	-0.449195	-0.204910
C	-4.293151	-0.407266	-0.181501
C	-4.582553	-1.691673	-0.751688
C	-5.393023	0.353986	0.335564
C	-5.853730	-2.182725	-0.798000
H	-3.791807	-2.275593	-1.208493
C	-6.669499	-0.138516	0.304415
H	-5.198835	1.327471	0.763154
C	-6.919962	-1.410681	-0.262810
H	-6.086898	-3.140990	-1.248471
H	-7.491521	0.439859	0.716864
O	-8.122283	-1.944303	-0.333335
H	-8.807560	-1.362491	0.039214
O	-3.492560	2.408410	0.149239
C	2.134250	-0.015059	-0.111979

C	2.576635	-0.377691	1.318859
C	3.617892	-1.482888	1.247044
C	4.762277	-1.047672	0.347564
C	4.234539	-0.664137	-1.033196
H	1.683533	-0.908830	-0.564322
O	3.264326	0.379180	-0.880317
C	5.333217	-0.161869	-1.966700
H	4.863980	0.304733	-2.837121
H	5.924711	0.609357	-1.446728
O	6.121240	-1.221253	-2.433356
H	6.490645	-1.677145	-1.659849
H	3.755192	-1.536182	-1.501694
O	5.703965	-2.082487	0.189974
H	5.947539	-2.386482	1.079671
H	5.232366	-0.156404	0.797661
O	4.172340	-1.768161	2.507422
H	3.458426	-2.088472	3.079345
H	3.147684	-2.379755	0.809730
H	3.033953	0.506081	1.786758
O	1.480876	-0.854385	2.076057
H	1.122588	-0.124485	2.598887

Daidzein -4'-O$^-$ anion

C	-3.58284800	-1.12017900	-0.13745200
C	-2.22613700	-0.77890200	-0.13734400
C	-4.51894100	-0.11730800	0.03901500
C	-1.79991400	0.54150400	0.03269200
C	-4.11933300	1.21993300	0.21571400
C	-0.00458100	-1.47303600	-0.32406500
C	-0.35949600	0.89211900	0.05577300
C	-2.77450500	1.53110700	0.21198000
H	-4.87110600	1.99430500	0.35321300
C	0.56263100	-0.25761200	-0.09720400
H	0.57621400	-2.36447200	-0.52102200
H	-2.42479400	2.54980400	0.34781700
O	-5.83720500	-0.47364200	0.03527200
H	-6.36804700	0.32347900	0.16890800
O	-1.34176000	-1.77594400	-0.31725100
C	2.01209600	-0.11927600	0.00126300
C	2.80469000	-1.18125700	0.49744400
C	2.70787300	1.02588900	-0.45615500
C	4.17764600	-1.13096000	0.52040400
H	2.30187000	-2.05659300	0.91262800
C	4.08101300	1.09158300	-0.44720100
H	2.13403100	1.86718600	-0.83304800
C	4.92489700	0.01345500	0.03226100
H	4.75661500	-1.95276100	0.93597700
H	4.59088700	1.98096800	-0.81104300
O	6.17533900	0.06773400	0.04012100
O	-0.01090500	2.05510500	0.20153500
H	-3.88998200	-2.15030800	-0.27360800

Daidzein -4'-O• radical

C	-3.55793800	-1.10902700	-0.16688100
C	-2.21218400	-0.76405500	-0.12664500
C	-4.49965800	-0.10228300	0.00205000
C	-1.78153900	0.54841300	0.07396300
C	-4.09808600	1.23071300	0.20713800
C	-0.00396700	-1.50492600	-0.25535300
C	-0.34692600	0.88296300	0.11143500
C	-2.75496200	1.54245700	0.24057300
H	-4.84914100	2.00542100	0.33762300
C	0.55179700	-0.28542900	-0.05030600
H	0.57927400	-2.40209100	-0.43021900
H	-2.41276600	2.56033900	0.39682800
O	-5.80244700	-0.46319300	-0.04036500
H	-6.36081800	0.31658000	0.09010600
O	-1.31628300	-1.78009200	-0.30030800
C	2.01293400	-0.15055300	-0.00336000
C	2.80759200	-1.20387500	0.52273200
C	2.65566500	1.01262400	-0.50347400
C	4.17298900	-1.12867800	0.52810000
H	2.31465600	-2.06808100	0.95955400
C	4.02129200	1.10575400	-0.51136000
H	2.04640800	1.82354500	-0.88127600
C	4.86056800	0.03687300	-0.00054200
H	4.78998100	-1.92124400	0.93870000
H	4.52899200	1.98098300	-0.90346600
O	6.10337200	0.11406400	-0.00724800
O	0.04821500	2.02600700	0.27414000
H	-3.86490200	-2.13556100	-0.32603900

Daidzein -7-O$^-$ anion

C	-3.66134200	-1.10346100	-0.16298100
C	-2.33273100	-0.76552400	-0.12937900
C	-4.69397900	-0.10461900	0.01412400
C	-1.86127400	0.55225900	0.07290300
C	-4.18966500	1.25333600	0.22091200

C	-0.11513400	-1.52042000	-0.27975900
C	-0.45871200	0.87085000	0.10407200
C	-2.85853200	1.54893500	0.24529000
H	-4.94073200	2.02758200	0.35605000
C	0.43844100	-0.30938800	-0.06211400
H	0.47356200	-2.41494900	-0.46492300
H	-2.50576900	2.56555400	0.40093400
O	-5.91156500	-0.36858600	-0.00817300
O	-1.42463600	-1.79587900	-0.32020700
C	1.90950500	-0.17686500	-0.01098800
C	2.71024900	-1.18649500	0.54176000
C	2.55752700	0.95376300	-0.52766900
C	4.09744100	-1.10318200	0.54917100
H	2.23204400	-2.04943900	0.99745900
C	3.94419700	1.05103400	-0.51586200
H	1.95547600	1.76287100	-0.92091600
C	4.71886300	0.02199100	0.01558900
H	4.71024200	-1.88752900	0.98176500
H	4.42958300	1.93690800	-0.92317100
O	6.08876300	0.07500100	0.04742200
H	6.36347100	0.92184000	-0.33000100
O	-0.00152600	2.01178100	0.25348800
H	-3.95530100	-2.13466100	-0.32784700

Daidzein -7-O• radical

C	-3.66379100	-1.11508400	-0.16921300
C	-2.32300400	-0.79234200	-0.14936900
C	-4.65366000	-0.08192600	0.03793800
C	-1.88803700	0.52634100	0.06517300
C	-4.17855900	1.27749200	0.26082900
C	-0.11268000	-1.52432800	-0.32014900
C	-0.44364800	0.84950200	0.08082400
C	-2.84420100	1.55468000	0.27034400
H	-4.93376000	2.04058200	0.41448900
C	0.44487200	-0.31370900	-0.09610500
H	0.47137100	-2.41809600	-0.50947500
H	-2.45923500	2.55611300	0.43208300
O	-5.87146200	-0.35256400	0.02474800
O	-1.43060600	-1.80321100	-0.35267000
C	1.91482200	-0.17280200	-0.03128800
C	2.69852700	-1.16311400	0.57649400
C	2.56274500	0.93618900	-0.58689500
C	4.08142300	-1.07232100	0.60896800
H	2.21412900	-2.00973200	1.05585300
C	3.94744400	1.03841900	-0.55526000
H	1.97780900	1.72636400	-1.04155500
C	4.71181600	0.03371200	0.03825900
H	4.68896400	-1.83399300	1.08521800
H	4.43733300	1.90497100	-0.99345200
O	6.06841200	0.08146900	0.10007700
H	6.37831200	0.90098600	-0.31055400
O	-0.05429900	1.99701900	0.23055200
H	-3.99568400	-2.13315600	-0.33757000

Daidzein

C	-3.62498100	-1.09375400	-0.17011000
C	-2.27323600	-0.76299300	-0.14412300
C	-4.55502200	-0.08319700	0.02749000
C	-1.83107200	0.54430400	0.07010300
C	-4.14050200	1.24362200	0.24691700
C	-0.07019800	-1.51031500	-0.31410300
C	-0.39257200	0.86686200	0.09243800
C	-2.79441000	1.54308700	0.26507900
H	-4.88362000	2.02214200	0.39948600
C	0.49223800	-0.30388000	-0.08941200
H	0.50736600	-2.40800000	-0.50512500
H	-2.44032800	2.55550900	0.43064200
O	-5.86381100	-0.43174600	-0.00135000
H	-6.41004900	0.35313400	0.14725800
O	-1.39180300	-1.78094700	-0.34587400
C	1.96370500	-0.17136700	-0.02806700
C	2.74544700	-1.16629600	0.57409700
C	2.61584300	0.93600800	-0.58192100
C	4.12951800	-1.08297700	0.60161100
H	2.25816300	-2.01173200	1.05259200
C	4.00140400	1.03143300	-0.55456600
H	2.03151200	1.73049600	-1.02957600
C	4.76310700	0.02126300	0.03238700
H	4.73480700	-1.84948400	1.07316000
H	4.49421600	1.89731300	-0.99127300
O	6.12188900	0.06326200	0.08847500
H	6.43054600	0.88443600	-0.31951000
O	0.01427700	2.00826200	0.24825100
H	-3.94266000	-2.11525200	-0.34058000

daidzein cation radical

C	-3.59733600	-1.11439500	-0.11248500
C	-2.26559200	-0.75142000	-0.08112500

C	-4.54934000	-0.09671500	-0.00293200
C	-1.82729300	0.56632800	0.05078100
C	-4.14716600	1.24505700	0.13236600
C	-0.06633200	-1.54260000	-0.16052400
C	-0.39951700	0.89476400	0.07996500
C	-2.80448800	1.56549800	0.15817600
H	-4.89791800	2.02533800	0.21761800
C	0.51568700	-0.29148100	-0.02351800
H	0.50131200	-2.45658100	-0.29668100
H	-2.47438600	2.59374300	0.26335100
O	-5.83387100	-0.47331400	-0.03491300
H	-6.42355900	0.29187700	0.04583600
O	-1.35014400	-1.78267200	-0.19642300
C	1.94492200	-0.17737300	-0.00573700
C	2.77582700	-1.30047700	0.32122000
C	2.59388700	1.06290400	-0.31848000
C	4.13574800	-1.20405500	0.32892900
H	2.32715400	-2.24008600	0.62273500
C	3.95852300	1.16294600	-0.32690700
H	1.98678600	1.92369700	-0.56111700
C	4.74934600	0.03437200	-0.00285900
H	4.77286500	-2.03917400	0.59772400
H	4.43907000	2.10211900	-0.58639000
O	6.06586000	0.05855700	0.01576700
H	6.42365300	0.93490900	-0.21021500
O	0.01125200	2.03405600	0.20359400
H	-3.90102500	-2.14897500	-0.21829000
